# Delayed management of secondary normal pressure hydrocephalus after cerebrospinal fluid external drainage

**DOI:** 10.1186/2045-8118-12-S1-P35

**Published:** 2015-09-18

**Authors:** Vincent Meyer-Bisch, Laurent Gergele, François Vassal, Benjamin Pommier, Christian Auboyer, Jêrome Morel, Romain Manet

**Affiliations:** 1 Department of Neurosurgery, University hospital of Saint-Etienne, France; 2 Department of Anesthesiology and Intensive Care, University Hospital of Saint-Etienne, France

## Introduction

External drainage (ED) of cerebrospinal fluid (CSF) is often used during management of brain insult. In absence of robust data and consensual guidelines, weaning ED remains challenging. The objective of the study was to assess the proportion of secondary normal pressure hydrocephalus (NPH), being treated with delay after ED weaning and to evaluate the resulting morbidity.

## Methods

All adult patients admitted in intensive care unit for brain insult between 1st january 2010 and 31st december 2013, managed with ED, were included in this retrospective monocentric observationnal study. Rate and delay of shunt surgery after ED weaning were analysed in the light of neurological prognostic assessed by modified Rankin scale (mRS). Literature review. National numeric survey adressed by email in all french neurosurgical center.

## Results

Among the 144 patients (57.6% of men) studied, 39% of the survivors developped a secondary NPH. Among them, 62, 5% were shunted early (from day 1 to day 7 after ED weaning) and 37.5% were treated with a delay (from 8 to 420 days after ED weaning). Identified risk factors of failure of the weaning test were: severity of initial neurological status (Glasgow score 9.5 vs 11), long delay between insult and ED insertion (226hrs vs 67hrs), low CSF outflow through ED (137mL/24h vs 194 mL/24h), high mRS at the moment of ED weaning (5 vs 4). Mean benefit of shunt insertion was 1 point of mRS in this population. Literature review found no evidence based data or guidelines to wean ED. National survey confirm the heterogeneity of ED weaning management.

## Conclusions

This study confirms the imprecision of ED weaning in absence of guidelines and robust data, resulting in delayed management of secondary NPH in more than one in three patients. Methods of ED weaning should be more studied and probably up-graded.

